# Multiple Sclerosis and Obesity: Possible Roles of Adipokines

**DOI:** 10.1155/2016/4036232

**Published:** 2016-09-18

**Authors:** José de Jesús Guerrero-García, Lucrecia Carrera-Quintanar, Rocío Ivette López-Roa, Ana Laura Márquez-Aguirre, Argelia Esperanza Rojas-Mayorquín, Daniel Ortuño-Sahagún

**Affiliations:** ^1^U.M.A.E. Hospital de Pediatría, C.M.N.O., Instituto Mexicano del Seguro Social, Guadalajara, JAL, Mexico; ^2^Instituto de Investigación en Ciencias Biomédicas (IICB), CUCS, Universidad de Guadalajara, Guadalajara, JAL, Mexico; ^3^Departamento de Farmacobiología, CUCEI, Universidad de Guadalajara, Guadalajara, JAL, Mexico; ^4^Unidad de Biotecnología Médica y Farmacéutica, Centro de Investigación y Asistencia en Tecnología y Diseño del Estado de Jalisco A. C., Guadalajara, JAL, Mexico; ^5^Departamento de Ciencias Ambientales, Instituto de Neurociencias, CUCBA, Universidad de Guadalajara, Guadalajara, JAL, Mexico

## Abstract

Multiple Sclerosis (MS) is an autoimmune disorder of the Central Nervous System that has been associated with several environmental factors, such as diet and obesity. The possible link between MS and obesity has become more interesting in recent years since the discovery of the remarkable properties of adipose tissue. Once MS is initiated, obesity can contribute to increased disease severity by negatively influencing disease progress and treatment response, but, also, obesity in early life is highly relevant as a susceptibility factor and causally related risk for late MS development. The aim of this review was to discuss recent evidence about the link between obesity, as a chronic inflammatory state, and the pathogenesis of MS as a chronic autoimmune and inflammatory disease. First, we describe the main cells involved in MS pathogenesis, both from neural tissue and from the immune system, and including a new participant, the adipocyte, focusing on their roles in MS. Second, we concentrate on the role of several adipokines that are able to participate in the mediation of the immune response in MS and on the possible cross talk between the latter. Finally, we explore recent therapy that involves the transplantation of adipocyte precursor cells for the treatment of MS.

## 1. Introduction

The prevalence of immune-mediated diseases has increased in recent years. In parallel, changes in dietary habits, which promote high-fat, high-sugar, and high-salt foods, have led to an obesity epidemic over the past years [1, 2]. According to the World Health Organization (WHO), approximately 35% of the world population is estimated to have overweight (Body Mass Index [BMI], 25–30 kg/m^2^) or obesity (BMI > 30 kg/m^2^) [3]. Therefore, it is reasonable to think about a possible correlation among these realities. In fact, obesity is considered a chronic state of low-grade inflammation that has been implicated as a proactive factor in several chronic autoimmune inflammatory disorders [4, 5].

Multiple sclerosis (MS) is an autoimmune disorder of the Central Nervous System (CNS), which is mainly characterized by selective and coordinated inflammatory destruction of myelin, with damage to the axon. It is a chronic and progressive inflammatory disease caused by an interaction of genetic and environmental risk factors. MS mainly affects young people with onset usually at the age of 20–50 years and a mean age-of-onset of 30 years, although the disease may also develop in childhood and after the age of 60 years [6]. Childhood and adolescence are thought to be a critical period of susceptibility to promoting factors.

This autoimmune disease has already been associated with several environmental factors, such as diet and obesity [7–10]. Obesity is a well-known risk factor for multiple conditions, including cardiovascular risk and metabolic disorders, as metabolic syndrome and insulin resistance [11], and has also been associated with a nonfavorable course of several autoimmune diseases [2]. Concomitant with the rise of MS is the increased prevalence of children with overweight and obesity in human population over recent decades [3].

Several studies have reported an increase in the prevalence of overweight and obesity among patients with MS [12], although some reported higher frequencies of patients with MS with a lower BMI [13] and some others reported similar rates of overweight and obesity in patients with MS and in general population [14]. Numerous publications have reported an association between MS and higher BMI in youth and early life [15–19]. Obesity in female patients with MS at the time of diagnosis is associated with a relapsing course at disease onset [20] and is also associated with a greater risk of depression, lower functional capacity, and worse self-rated health status among patients with MS [21, 22]. However, a direct relationship between MS and obesity remains inconclusive [23].

A reported cooccurrence of obesity in patients with MS may simply reflect increased obesity in the population or it may be secondary to the harmful effects of the disease. However, a positive correlation between BMI and disability evaluated by the Expanded Disability Status Scale (EDSS) was reported recently [24]. To date, scarce information is available on the association of metabolic comorbidities and disability in individuals with MS. One study [25] showed that vascular comorbidities, such as diabetes, hypertension, hypercholesterolemia, and peripheral vascular disease, were independently associated with an increased risk for disability. In contrast, another study reported that worsening disability was associated with higher low-density lipoprotein cholesterol, total cholesterol, and triglycerides in patients with MS [26]. Additionally, an association exists between disability and Oxidative Stress (OS) in patients with MS [27]. What is becoming clear is that obesity influences both disease progress and treatment response. Additionally, a couple of recent studies in large populations clearly associate obesity in early life, during adolescence [28], and during childhood and/or early adulthood [29] with a higher MS risk, providing further solid evidence strongly suggesting that obesity is also relevant by means of its causal role in MS etiology.

The possible link between MS and obesity has become even more interesting in recent years since the discovery of the remarkable properties of adipose tissue. Adipose tissue has the ability to expand in response to chronic caloric excess as an adaptive response. In individuals with obesity, adipose tissue can constitute up to 50% of total body mass. Since the exciting finding of the secretory properties of adipose tissue, the relationship between obesity and autoimmunity and the understanding of the underlying mechanisms have become of major interest. Indeed, fat tissue, in addition to its function as an energy storage site, has been found to produce a wide variety of soluble mediators denominated “adipokines,” which are involved in the regulation of numerous physiological functions, such as the regulation of energy balance, insulin sensitization, and the immune response [2]. Initially identified for their metabolic and appetite regulation activities, adipokines are known to be involved in various processes, including immunity and inflammation. Because of their proinflammatory action, these molecules contribute to the so-called “low-grade inflammatory state” in subjects with obesity [30].

Obesity is associated with self-directed tissue inflammation, in which local or systemic factors other than infectious agents activate the cells of the innate immune system. During the development of obesity, adipocytes undergo considerable differentiation and expansion in order to store lipids [31]. However, immune cell infiltration and activation within adipose tissue are a major source of proinflammatory cytokines, which impair adipocyte function in chronic obesity. Thus, alterations in adipose tissue and the development of chronic inflammation are the hallmarks of obesity and are at least partially responsible for the induction of insulin resistance.

On this basis, the aim of this review was to present, integrate, and discuss recent evidence on the possible link between obesity as a chronic inflammatory state and the pathogenesis of MS as a chronic autoimmune and inflammatory disease. First, we briefly describe the main cells involved in MS pathogenesis, both from neural tissue, including glial cells (such as microglial cells, oligodendrocytes, and astrocytes) and neurons, as well as cells from the immune system such as different T cell lineages, CD4+ (such as Th1, Th2, and Th17), CD8+, and Treg (CD4+CD25+FoxP3+), and also including a new participant, the adipocyte, a cell from the adipose tissue, focusing on their roles in MS. Second, we concentrate on the role of several adipokines that are able to participate in the mediation of the immune response in MS and on the possible cross talk between these. Finally, we explore recent therapy that involves the transplantation of adipocyte precursor cells for the treatment of MS.

## 2. Cells Involved in MS

### 2.1. CNS Cells

#### 2.1.1. CNS: Microglia

Microglia constitute the resident macrophages of the CNS [40] and are involved in immune processes as such as Antigen Presenting Cells under pathological conditions [41]. Microglia express several immunological markers, such as MHC I, MHC II, CD40, CD11b, Fc receptors I–III, Complement Receptors (CR1, CR2, and CR4), *β*2 integrins, CD80, CD86, and IntraCellular Adhesion Molecule-1 (ICAM-1) [42]. Dual roles of macrophages have been described in human neuroimmune diseases such as MS. Whereas proinflammatory macrophages secrete harmful molecules to induce disease development, anti-inflammatory macrophages produce beneficial mediators to promote disease recovery [43].

In experimental autoimmune encephalomyelitis (EAE) in mice, an animal model for MS, the expressions of MHC II and CD86 are increased (Table 1). This suggests that microglia participate in the antigen presentation to the T cells that migrate into the CNS. In addition, in MS lesions, microglia and CNS-infiltrating macrophages interact with oligodendrocytes by their expression of VCAM-1 [44] and with phagocyte myelin and axon debris, via HLA-DR, contributing to the demyelination process [45].

The activation of microglia in MS is a dynamic process, because there is an intermediate state in all preactive and remyelinating lesions that differ from microglia profiles in actively demyelinating lesions [46]. Antigen-activated microglia produce proinflammatory cytokines, such as InterLeukin-1beta (IL-1*β*), Tumor Necrosis Factor alpha (TNF-*α*), and IL-6 [42]. A subset of CD4+ T cells that produces IL-17 or IL-17 and InterFeroN gamma (IFN-*γ*) migrates to the CNS prior to the onset of EAE clinical symptoms (revised forward). This event coincides with microglia activation and their production of IL-1*β*, TNF-*α*, and IL-6 in the CNS [47]. Treatment with Glatiramer Acetate (GA) prevents interaction between T cells and microglia and contributes to the amelioration of MS severity in patients [48]. These facts support that, in the pathogenesis of MS, both peripheral immune system cells and CNS resident cells are involved from disease onset.

In a recent study, Michels et al. demonstrated, in an animal model of sepsis, that costimulation of microglia through CD40-CD40L is associated with brain inflammation, oxidative damage, and Blood Brain Barrier (BBB) dysfunction [49]. In addition, soluble CD40L has been associated with the complications of obesity, such as cardiovascular disease, insulin resistance, and chronic inflammation [50]. CD40 is a transmembrane receptor expressed in a variety of cell types including microglia in the CNS [49]. The interaction between CD40 and its ligand (CD40L), expressed by T cells, induces costimulation in the presenting antigen and enhances the production of cytokines, chemokines, matrix metalloproteinases, growth factors, and adhesion molecules, mainly through NF-*κ*B [51]. Also, the presence of CD40L plus IFN-*γ* induces ERK1/2-mediated Monocyte Chemoattractant Protein-1 (MCP-1) and p38-mediated IFN-Inducible Protein-10 (IP-10) production by microglia. These chemokines have been associated with CNS pathologies, including MS [52]. It is possible that CD40/CD40L interactions between microglia/macrophages and T cells are involved in MS pathogenesis; thus, future studies should be conducted on this aspect.

Regarding microglia cells participation in axonal damage, activated microglia releases glutamate, which induces excitotoxicity and contributes to damage. Blockade of glutamate release decreases neuronal death [53]. In this respect, higher levels of glutaminase, produced by macrophages and microglia, are found in MS lesions, and these levels are correlated with axonal damage in animals. In addition, GLutamate Transporter-1 (GLT-1) has low expression levels in active lesions, and Glutamine Synthetase (GS) and Glutamate DeHydrogenase (GDH) are absent in active and chronic, silent MS lesions [54]. This glutamate unbalance leads to neural death and increases CNS damage and disease severity.

Microglia can not only be involved in the inflammatory and neurodegenerative process. It is possible to induce a neuroprotective state in the microglia through a stimuli with IL-4 and IL-25 [55]. This neuroprotective state can induce oligodendrogenesis through its activation by IL-4 and IFN-*γ* and a later release of Insulin-Like Growth Factor-1 (IGF-1) [56]. Additionally, intraparenchymal microglia produce a Macrophage-Derived Chemokine (MDC/CCL22), which could induce Th2 cell migration into the CNS and mediate Th1-migration [57]. Furthermore, Programmed Death-Ligand-1 (PD-L1) acts as an inhibitory costimulatory molecule that is expressed by microglia after stimulation with IFN-*γ* [58] or IL-6 [59], and CNS-infiltrating T cells exhibit overexpression of their receptor Programmed Death-1 (PD-1) at the EAE peak and prior to remission. In this regard, microglia can inhibit CD4+ T cell proliferation and Th1 cell differentiation in a PD-L1-dependent manner [41]. Therefore, microglia can play a dual role in the pathogenesis of MS, depending on the cytokine microenvironment, where it can perform inflammatory functions that promote axonal damage and neural death or anti-inflammatory functions that inhibit cell migration into the CNS.

*In vitro*, Peroxisome Proliferator-Activated Receptor gamma (PPAR-*γ*), a regulator of adipocyte differentiation implicated in obesity [60], is strongly upregulated following demyelination mediated by antibodies directed against the Myelin Oligodendrocyte Glycoprotein (MOG) in the presence of complement [61]. Pioglitazone, an agonist of PPAR-*γ* that modulates the transcription of insulin-sensitive genes [62], partially protects aggregates from anti-MOG demyelination, and it appears to be linked with an inhibition of glial cell proinflammatory activities following anti-MOG-induced demyelination [61]. Additionally, a similar effect to that of pioglitazone can be provided by nutraceutics, which can modulate PPAR-*γ* signaling and which can be employed as a complimentary treatment for obesity-related disorders [63]. Activation of PPAR-*γ* signaling regulates adiponectin production, a protein with anti-inflammatory properties trough the inhibition of proinflammatory cytokines and a protective role against insulin resistance [64]. PPAR-*γ* agonists can modulate EAE by the inhibition of NO, TNF-*α*, IL-1*β*, IL-6, and MCP-1 production from microglia and astrocytes [65] and also by inhibiting IL-12 production and signaling and Th1 differentiation [66]. In addition, it has been demonstrated that Steroid Receptor Coactivator-3 (SRC-3) inhibits adipocyte differentiation [67] and controls the expression of PPAR. SRC-3 deficiency attenuates EAE severity through the upregulation of PPAR-*β* in the CNS and the subsequent microglia-alternative activation, which modulates neuroinflammation and promotes remyelination [68]. Together, these data suggest that the PPAR agonist could modulate the demyelination process in EAE and MS.

On the other hand, microglia express estrogen receptor-*β*, and their signaling reduces NF-*κ*B expression, as well as NF-*κ*B-induced gene-inducible Nitric Oxide Synthase (NOS) in microglia and CD3+ T cells [69]. It has been demonstrated that T cells induce microglia activation through C/EBPbeta, but not NF-*κ*B, in EAE, but only in female mice. This is one of the mechanisms that could explain why MS is more prevalent in women [70]. Also, estrogen pretreatment in EAE mice enhanced the frequency of regulatory B cells and anti-inflammatory M2 microglia [71]. These evidences suggest that estrogen treatment promotes neuroprotection through the inhibition of T cell and macrophage recruitment into the CNS and microglia activation, which may represent a benefit for MS treatment.

#### 2.1.2. CNS: Oligodendrocytes

Oligodendrocytes are the myelinating cells of the CNS. Both neurons and oligodendrocytes are affected during brain damage in acute and chronic neuroinflammation, leading to demyelination processes [102]. In MS, oligodendrocytes are damaged by different processes and their number decreases. Oligodendrocyte Precursor Cells (OPC) are present in both normal CNS and MS lesions [103]. There is evidence indicating changes in the mechanisms of migration of OPC and myelinating oligodendrocytic activity. In this sense, Bin et al. found that netrin-1, an inhibitor of Oligodendrocyte Precursor Cell migration, is secreted by myelinating oligodendrocytes in MS lesions [104]. In addition, Sádaba et al. demonstrated the presence of IgM and IgG in demyelinating lesions localized in axons and oligodendrocytes from the autopsies of patients with MS [105]. However, the presence of Platelet-Derived Growth factor alpha Receptor (PDGRalphaR) and NG2 OPC in active MS lesions, as in remyelinated MS tissue, indicates that these cells are potential sources of remyelinating oligodendrocytes in active lesions [106]. Furthermore, the expression of CD200 in neurons, oligodendrocytes, and reactive astrocytes in MS chronic plaques and the interaction with its receptor CD200R suppress immune activity [107], and this affects neuron-microglia and glia-glia interactions.

In an attempt to prevent this damage, it has been reported that oligodendrocytes stimulated with IFN-*γ*, a proinflammatory cytokine, could be protected. Pancreatic Endoplasmic Reticulum Kinase (PERK) signaling can be activated in oligodendrocytes in the presence of IFN-*γ* in MS lesions [108]. The activation of PERK signaling pathway in oligodendrocytes attenuates EAE severity by a reduction of oligodendrocytic apoptosis and demyelination [109], indicating that activation of an integrated stress response could ameliorate MS severity.

#### 2.1.3. CNS: Astrocytes

Astrocytes are the most abundant type of cells in the CNS. They participate in maintaining normal brain function and are in constant communication with neurons, oligodendrocytes, and Endothelial Cells. Also, astrocytes are involved in angiogenesis, neurogenesis, synaptogenesis, and axonal remodeling [110]. However, B7-1 (CD80) or B7-2 (CD86) is not expressed by astrocytes and CNS Endothelial Cells (EC) in EAE. This suggests that neither type of these cells can induce costimulatory signals via B7 molecules and that they are incapable of acting as Antigen Presenting Cells (APC) [111].

It has been demonstrated that astrocytes are involved in the progress of MS through several mechanisms, which include the following: releasing cytotoxic factors, inhibiting axonal remyelination, and contributing to axonal mitochondrial dysfunction [112]. In this regard, neuregulin production, an oligodendrocytic growth factor, is depleted in astrocytes [113], leading to the decrease of the oligodendrocytic maturation ability. Also, CD24 is expressed in both immune and CNS cells. Liu et al. demonstrated that CD24 expression enhanced costimulatory activity of astrocytes with T cells and increased EAE severity [114]. Furthermore, astrocytes and microglia can express inducible NO Synthase (iNOS) in EAE lesions [115], which contributes to axonal damage [116]. These data demonstrate the relevant contribution of astrocytes to MS pathogenesis and progression.

#### 2.1.4. CNS: Neurons

The immune-mediated destruction of CNS myelin and oligodendrocytes has traditionally been considered the primary damage in patients with MS; however, the irreversibility of the neurological disability corresponds to cortical damage, driven by either neuronal loss induced by retrograde degeneration from white-matter lesions or as a direct consequence of the localization of demyelinated plaques within the cortex [117]. Main damage to neurons derives from the demyelination process, which affects myelin sheaths as well as the oligodendrocyte itself [118].

The profound oxidative injury observed in MS lesions appears to be related to mitochondrial impairment in damaged axons [119]. Consistently, synaptic loss comprises a characteristic feature of gray matter lesions (particularly in type I lesions) [120], and neurite density is proportionally reduced with increased meningeal inflammation [121]. Neurons can also interact with CD4+ T cells and induce its differentiation into CD25+ TGF-beta1+ CTLA-4+ FoxP3+ Treg through the interaction between the B7-CD28 and TGF-beta1-TGF-beta receptor signaling pathways. These Treg suppress encephalitogenic T cells in a CTLA-4 manner [122]. This data indicates that neurons can execute mechanisms that attempt to reverse the damage in the demyelination process.

### 2.2. Immune System Cells

#### 2.2.1. Immune System: T Cells

T cells are a highly heterogeneous population of cell subtypes that mediate adaptive immunity and specific tolerance [123]. There are several subpopulations of T cells that differentiate upon encountering antigens in the peripheral lymphoid organs [124]. In MS, the role of T cells is crucial to trigger the immunopathological processes that culminate in demyelination and subsequent damage to oligodendrocytes and axons [125]. Several studies demonstrate that pathogenic T cell response against myelin antigens is followed by a neurodegenerative process [126]. However, it is difficult to establish a correlation between CNS-infiltrating and peripheral T cell subsets. In this respect, it is necessary to consider the possible existence of subgroups of patients, depending on treatment and time of disease evolution [127] to identify the correlations of these subgroups with variations in the T cell subsets present in MS pathogenesis.

#### 2.2.2. Immune System: CD4+ T Cells

CD4+ T cells could play a central role in the pathogenesis of the EAE model. These cells cross the BBB and cause axonal damage and neuronal death [128]. Within the CNS, microglial cells interact with T cells by antigen presentation and the production of injurious or neurotrophic outcomes in their vicinity [129]. Additionally, activation of memory CD4+ T cells is associated with the exacerbation of MS; in addition, activation of memory CD8+ T cells (see later) reflects a dysregulation of the immune system in patients with MS [130]. Full activation and posterior proliferation of T cells usually require an Antigen Presenting Cell- (APC-) derived costimulatory signal, which is delivered via B7/CD28 [131]. However, the T cell proliferative response in MS is relatively independent of B7-CD28 costimulation [132–134].

Additionally, autoreactive T cells against myelin components can be detected in both the serum and CerebroSpinal Fluid (CSF) of patients with MS [135]. Burns et al. described that 84% of the MBP-reactive T cells are memory T cells in peripheral blood [136] but, according to Chou et al., only 37% of T cells isolated from patients with MS are MBP- or PLP-reactive in LCR [137]. Furthermore, CD4+ T cells in CSF are predominantly helper inducers, and their increase may contribute to local autoimmune process in the CNS [138]. Taken together, these studies prove that T cells reactive to myelin play an important role in the pathogenesis of MS.

#### 2.2.3. Immune System: Th1 and Th2

Th1 cells secrete high levels of IFN-*γ* and IL-12, which are considered proinflammatory cytokines. On the other hand, Th2 cells secrete high levels of anti-inflammatory cytokines, mainly IL-4, IL-5, IL-9, and IL-13, and promote the humoral response [139].

Originally, MS was considered as an autoimmune disease, driven by Th1 cells. This was supported by three facts: (1) CD4+ T cells isolated from CNS from mice with EAE express detectable levels of IFN-*γ* messenger RNA (mRNA) [140]; (2) IL-12p40-defective (IL-12p40−/−) mice are resistant to EAE induction, because IL-12 is a requirement for naïve T cell differentiation in Th1 cells [141]; and (3) patients with MS exhibited exacerbation during treatment with IFN-*γ* [142]. However, several studies suggest that there is a dysregulation in the balance between Th1 and Th2 cytokine profiles in MS [143].

The Transforming Growth Factor-Beta (TGF-*β*) inhibitor Smad7 is an important negative modulator that regulates the strength of TGF-*β* signaling [144] and it is upregulated in peripheral CD4+ T cells from patients with MS during disease relapse, but not during remission. This Smad7 expression correlates with T-beta responses, suggesting that Smad7 drives Th1 differentiation and regulates the inflammatory cellular response [145]. Additionally, disease progression can be altered by a hormonal component that modifies T cell number and cytokine secretion. In this sense, CD4+ IFN-*γ*-producing cells fluctuate with MS relapses: declining during pregnancy, in women with MS, and continuing to decline after parturition, in women with relapses. In contrast, these cells rise, or remain stable, in women with nonrelapsing MS or healthy pregnant women [146].

In contrast, Th2 cells have been described as being protective in MS/EAE [147]. Th2 cells are able to suppress microglial activation via cell-to-cell contact [148]. Furthermore, treatment with GA, which shifts the cytokine profile from Th1 to Th2, is regularly utilized as immunomodulatory therapy for MS [149, 150]. This suggests that Th2 cells are activated by GA in the periphery, migrate into the CNS, and then produce the Th2 cytokine profile after local recognition of Myelin Basic Protein (MBP) [150]. In the case of other treatments, patients with MS treated with IFN-*β* showed downregulation of circulating T cells secreting IFN-*γ* and IL-4 [151].

However, despite the anti-inflammatory behavior of Th2 cells in autoimmune diseases, in a recent work, Planas et al. identified clonally expanded CD4+ T cells releasing Th2 cytokines in T cell infiltrate of pattern II brain autopsy lesions, and the authors argue that this subset possesses a pathogenic and not a protective role in MS [152].

It is noteworthy that Th1 and Th2 cells infiltrate adipose tissue which varies according to regional fat depot. Th1 cells are higher in Visceral Adipose Tissue (VAT) than in Subcutaneous Adipose Tissue (SAT) and peripheral blood in healthy individuals with overweight and obesity. However, these proinflammatory T cell frequencies in VAT are correlated with SAT and peripheral blood [153]. This suggests that peripheral T cell subsets could also be associated with adipose tissue-infiltrating T cells in patients with MS and with obesity.

#### 2.2.4. Immune System: Th17

The T-helper 17 (Th17) cells are a subset of CD4+ effector T lymphocytes that challenges the Th1/Th2 paradigm of the immune response. Th17 cells mainly secrete IL-17 and are currently recognized for their involvement in the pathogenesis of autoimmune diseases [154, 155].

In the CSF of patients with Relapsing-Remitting Multiple Sclerosis (RRMS), Th17 cell frequency is higher in the relapse than in the remission stage or than in other noninflammatory neurological diseases [156]. In acute lesions, the expression of Retinoic Acid-Related Orphan nuclear hormone Receptor (RORc) and the cytokine genes that participate in Th17 expansion are upregulated in acute autopsy lesions of patients with RRMS [157]. Because Th17 promotes BBB disruption and causes the increase of CD4+ T cell infiltration in the CNS [158], the presence of Th17 cells in the CNS is able to give rise to microglial activation and the increase of the proinflammatory cytokine microenvironment [47]. Furthermore, IL-17, a cytokine produced mainly by Th17 cells, induces higher production of glutamate, causing excitotoxicity [159]. This cytokine has been investigated in autoimmune diseases [160], including MS [161].

Th17 cells can be influenced by the cytokine microenvironment, being able to exhibit certain developmental plasticity by upregulating IFN-*γ* and T-bet with a decreasing expression of IL-17 and ROR*γ*t [162]. Additionally, Carbajal et al. demonstrated that plastic Th17 cells can also be mediators of demyelination and axonal damage, as well as of Th1, and can induce the classical features of MS independently of differentiation factors IL-23 and IL-12 [163]. These evidences indicate that there is a possible link between Th1- and Th17-mediated autoimmune demyelinating processes, which can provide a future, common therapeutic target for MS treatment.

The search for serum biomarkers that describe the behavior of MS includes the study of cytokines such as IL-17. However, it is difficult to correlate these levels with the evolution of the disease. Thus, the existence could be considered of subgroups of patients with specific characteristics, considering age, treatment, and time to disease progression [127]. In this respect, it would be possible for T cell subsets to also vary according to these subgroups in patients with MS.

Regarding treatment, IFN-*β* induces greater STAT1 activation in Th17 compared to Th1 because Th17 cells express higher levels of InterFeroN alpha/beta Receptor 1 (IFN-*α*/*β*R1). This result indicates that IFN-*β* can inhibit Th17 cell expansion [164]. Therefore, IFN-*β* suppresses Th17 differentiation* in vitro* by inhibiting Th17 cell-lineage markers RORc, IL-17A, IL-23, and CCR6 and by inducing IL-10 production [165, 166].

Recently, it was reported that obesity may predispose induction of Th17 cells, at least, in part, in an IL-6-dependent process, which exacerbates autoinflammatory diseases such MS in mouse models [167]. Paradoxically, IL-17 has also been shown to inhibit adipogenesis [168]. Therefore, the precise role of Th17 cells and IL-17 in the obesity-associated inflammatory conditions needs to be further clarified.

#### 2.2.5. Immune System: CD4+CD25+FoxP3+ Treg

Regulatory T cells (Treg) comprise specialized suppressor T cell population that restrains the pathogenic immune response [169], while CD4+CD25+FoxP3+ Treg maintain tolerance to self-antigens [170]. Treg mediate the autoimmune inflammation induced in EAE through Cytotoxic T Lymphocyte Antigen- (CTLA-) 4-dependent suppression of pathogenic T cells [122]. Posterior recovery correlates well with the accumulation of Treg, that are producers of IL-10 [171]. In MS, the Treg function is defective and this contributes to the pathogenesis of the disease [170]. Also, Treg are capable of migrating across the BBB under noninflammatory conditions but, in patients with RRMS, this ability is impaired [172]. This change contributes to breaking the homeostasis in the CNS and could facilitate the initiation of CNS inflammation.

The suppressor activity of T cells in the peripheral blood of patients with patients in remission-clinical-phase was first described by Huddlestone and Oldstone [173]. Since that time, controversial results have been reported concerning the presence of Treg during the fluctuating behavior of MS. In this regard, the frequency of CD4+CD25+FoxP3+ Treg was described as lower in patients with MS than in healthy controls [174, 175], but there was no correlation with clinical variables [174]. Bach et al. previously described that these cells are depressed in patients with acute exacerbation of MS [176]. Contrariwise, Dalla Libera et al. demonstrated that Treg are restored during an acute clinical attack [177]. This suggests that Treg are not involved in causing clinical relapses, but, rather, they can react to the inflammation in an attempt to restore the balance.

Regarding treatment, Praksova et al. described that both IFN-*β* and GA increase naïve T cells and decrease central memory T cells [175], but only Glatiramer Acetate (GA) increases Treg [175, 178]. However, this is in contrast with other works reporting that CD4+CD25+FoxP3+ Treg are increased in patients with MS treated with IFN-*β* [179, 180], without any significant effect on* FoxP3* gene expression after 6 months [180]. It has been described that lFN-*β* induces downregulation of CTLA-4 in Treg [179]. With respect to treatment effect, it has not been possible to date to establish any relationship between obesity and the use of lFN-*β* or GA [181].

On the other hand, glucocorticoid treatment restores impaired Treg function and increases IL-10 production in relapsing patients with MS [182]. Further, glucocorticoids modulate T-bet and STAT1 expression in mononuclear cells from patients with RRMS [183]. The effect of glucocorticoid treatment contributes to the decrease of neuroinflammation. However, the chronic use of glucocorticoids is associated with weight gain and increased obesity and related disorders [184, 185].

With respect to sexual hormones, these appear to be involved in the increase of Treg, as well as other T cell populations. It has been described that, during gestation, there is a physiological expansion of Treg that contribute to decreasing EAE manifestations and reducing CNS demyelination [186]. In addition, E2 and P2 enhance Treg function* in vitro* [187], leading to the suggestion that the use of E2 is capable of being a therapy for MS [188, 189] but that estrogens participate in metabolic functions and their use in humans produces the accumulation of subcutaneous fat [190] which could trigger obesity and metabolic disorders.

#### 2.2.6. Immune System: CD8+ T Cells

Because the study of the immunological basis involved in MS has focused mainly on CD4+ T cells as mediators of the disease [191, 192], the role of CD8+ T cells in MS is unclear to date. However, CD8+ T cells constitute the predominant T cell population in lesions in patients with MS and are oligoclonally expanded at the site of the pathology [192]. Furthermore, it has been recently demonstrated that CD8+ T cells engage in both roles: the pathogenic and the regulatory role in MS [192].

CD8+ T cells and CD4+ T cells exhibit different epigenetic signatures because they have distinct DNA methylation profiles [193]. It has been demonstrated that myelin-specific CD8+ T cells infiltrate the CNS and play a pathogenic role in EAE. In addition, the presence of CD4+ T cells is required for more severe disease and sustained neuroinflammation [194]. Furthermore, CD8+ T cells participate in myelin-specific CD4+ differentiation into CNS-infiltrating effector cells in secondary lymphoid organs [195]. Therefore, all of these evidences strongly suggest that disease severity depends on the cooperation between CD4+ and CD8+ T cells.

On the other hand, CD4+ T cells expressing Latency-Associated Peptides (LAP) suppress EAE depending on the TGF-*β* [196]. But not only CD4+ T cells can express LAP. CD8+ LAP+ Treg comprise a novel subset that has a regulatory function and that reduces the disease severity of EAE by TGF-*β*- and IFN-*γ*-dependent mechanisms [197].

In the same way that CD4+CD25+FoxP3+ does, CD8+CD28− Treg also regulate the balance between immunity and tolerance [174]. CD8+CD25+FoxP3+ T cells are IL-10 and TGF-*β* producers and downregulate costimulatory molecule expression in dendritic cells through a STAT3-mediated pathway, inducing a less efficient antigen presentation and inducing 2,3-dioxygenase (IDO) through STAT3 and cytotoxic T lymphocyte antigen-4-dependent mechanisms [198]. Furthermore, CD8+ T cells, with immunosuppressive functions, are induced in MS patients treated with GA, and they appear to be HLA-E restricted [192]. These latter data demonstrate how CD8+ T cells can play a regulatory role in the evolution of MS.

### 2.3. Adipose Tissue

#### 2.3.1. Adipose Tissue: Adipocytes

Although clinical evidence correlating obesity and MS is growing [9, 181], very little is known to date about the possible involvement of adipose tissue in MS pathogenesis as well as in cross talk among the tissues involved. Therefore, this constitutes a whole new field to be explored. It has been hypothesized that there is a link between metabolism and MS that involves proinflammatory mediators such as leptin, which promotes environmental conditions that in turn promote the loss of immune self-tolerance [199]. Additionally, human adipocytes express the costimulatory receptor CD40, suggesting that these cells contribute to obesity-related inflammation. Also, T cells regulate adipokine production through soluble factors and contact with adipocytes, involving CD40 [200].

The apparently passive role played by adipocytes, in pathophysiological terms, has been gradually substituted with a metabolically active performance, relevant to many biochemical mechanisms that may contribute to a chronic low-grade inflammatory status, which increasingly imposes itself as a key feature of obesity [181]. As natural regulatory T cells (nTreg) do, adipocytes also could play an important role in the immunopathogenesis of MS and in the associated inflammation. In fact, very recently, cells that are able to differentiate into adipocytes, as mesenchymal stromal cells, are being utilized in transplantation as therapy to treat autoimmune diseases, in particular MS (see the last part of this review).

## 3. Adipokines Involved in Cellular Cross Talk in MS

Clinical and experimental data, together with epidemiological studies, have suggested that the pathogenesis of MS might involve factors that link the immune system with the metabolic status [199]. Therefore, a very exciting field in recent investigation comprises the role of adipokines in the pathogenesis of MS. Some studies have reported increased levels of leptin, resistin [32], and visfatin and decreased levels of adiponectin in patients with RRMS in comparison with healthy controls [33], a profile also observed among subjects with obesity [201–203] (Table 2).

### 3.1. Adipokines

#### 3.1.1. Leptin

Leptin is an adipokine predominantly produced by adipose tissue and secreted into the circulation [204]. This adipokine is a regulator of body weight that promotes satiety and stimulates energy expenditure [205], and its levels correlate with the body adipose mass as well as with adipocyte size [206]. Leptin is upregulated by inflammatory mediators, such as TNF-*α*, IL-1 [207], and IL-6 [208]. In addition to its metabolic effects, leptin is also a potent modulator of immune responses [209]. Thus, patients who are congenitally leptin-deficient have a higher incidence of infection-related death due to dysfunctional immune response [210], with reduced numbers of circulating CD4+ T cells, impaired T cell proliferation, and cytokine release [211]. Leptin modulates immune response toward a proinflammatory profile and is found at the crossroads between inflammation and autoimmunity. Upsetting its balance may result in immunosuppressed condition, or, conversely, in a proinflammatory state, then facilitating the development of autoimmune diseases [2].

Leptin receptor (LepR) is expressed in CD4+, CD8+ [212, 213], Treg [214], and Natural Killer (NK) cells [215] and in monocytes/macrophages [212]. Because of the expression of their receptor, leptin induces differential effects in CD4+ T cells. Naïve T cells increased proliferation, whereas memory T cells were inhibited. Furthermore, leptin increases IFN-*γ* production and inhibits IL-4 production in memory T cells [216].

In patients with MS, LepR expression has been found significantly higher in CD8+ T cells and monocytes from patients in relapse phase than that observed in patients in remission (or in healthy controls). Moreover, exogenous leptin treatment sustains STAT3 phosphorylation, but only in monocytes from relapsing patients [76], suggesting that LepR might play a role in the modulation of clinical relapses during MS. A gene microarray analysis of Th1 lymphocytes from active MS lesions has shown elevated transcripts of many genes of the neuroimmune endocrine axis, including leptin [217]. Additionally, leptin serum levels are elevated in patients with MS before relapses and after treatment with IFN-*β* [85]. These data suggest that leptin and its receptor induce Th1 cell and cytokine environment and favor the induction of inflammation in MS.

Moreover, in a recent work, Yu et al. demonstrated that Th17 cell frequency is reduced in leptin-deficient (ob/ob) mice and that the administration of exogenous leptin restores the Th17 cell count. Additionally, leptin induced Th17 response through ROR*γ*t transcription in normal human T cells [218]. In the animal model of arthritis, leptin enhances Th17 cell response and exacerbates joint inflammation [219]. Thus, the findings suggest that leptin can induce an altered immune state and should contribute to neuroinflammation in MS through both Th1 and Th17 cell response.

Although there is evidence suggesting that leptin is associated with the development of CNS in mice [220, 221], it has been demonstrated in the animal model that ob/ob mice are resistant to EAE induction and that leptin-deficient (ob/ob) mice exhibit impaired cellular immune response, abnormal cytokine production, and impaired T cell proliferation [211]. Protection in ob/ob mice was associated with a progressive decline in the survival of autoreactive CD4+ T cells and reduced production of Th1 and Th17 cytokines. T cells demonstrated downregulation of Bcl-2, a survival protein, reduction in P-ERK1/2, and cell-cycle arrest associated with reduced degradation of cell-cycle inhibitor p27^kip1^. Additionally, there is impairment at the level of the nutrient-energy-sensing AKT-mTOR/S6 signaling pathway, which can be restored* in vivo* with leptin administration [222]. Taken together, the evidence allows the consideration of leptin and/or its receptor as important players in the pathogenesis of MS, as well as their consideration in therapeutic targets of the disease.

Matarese et al. found that leptin is increased in CSF and in the serum of patients with RRMS. These levels are related to IFN-*γ* production in the CSF and, inversely, with the proportion of Treg [35]. In this respect, several studies showed that Treg accumulated in normal adipose tissue [223–225].

Recently, the relationship between caloric restriction and survival in MS has been investigated. In an animal model, Piccio et al. established that caloric restriction and reduction of leptin serum levels can increase survival and lifespan through the reduction of inflammation, demyelination, and axonal injury [84]. At the same time, Longo and Fontana demonstrated that caloric restriction reduces inflammatory parameters and suggest that nutritional intervention improves the inflammatory response [226]. Caloric restriction has also been associated with an increase of the ghrelin, NeuroPeptide Y (NPY), which can moderate EAE [227]. All of the previous evidence indicates that leptin is able to participate as a link between metabolism and MS. So, it is necessary to find out the role of neurohormonal response in the ingestion of food, which is orchestrated by the hypothalamic circuitry in patients with MS.

#### 3.1.2. Adiponectin

Adiponectin (APN) is the most abundant circulating adipokine and is involved in metabolic diseases such as type 2 diabetes, metabolic syndrome, and related complications, especially cardiovascular diseases. Also, evidence indicates that APN is related to the severity of rheumatoid arthritis, systemic lupus erythematosus, and osteoarthritis [228]. Systemic and local levels of APN are elevated in patients with inflammatory and immune-mediated diseases [155, 168, 229].

APN exerts its effects through its receptors. AdipoR1 is expressed predominantly in skeletal muscle, AdipoR2 is found more abundantly in liver, and T-cadherin is mainly expressed in the cardiovascular system [230]. In this regard, APN exhibits anti-inflammatory activity on immune system cells [231]. Its functions include suppression of the proliferation of myelomonocytic precursor cells and the phagocytic activity of mature macrophages [232], regulation of monocyte-to-fibroblast transition [233], regulation of TNF-*α* and IFN-*γ* production in response antigen presentation [234], and the induction of IL-10 and IL-1 Receptor Antigen (IL-1RA) in monocytes, macrophages, and dendritic cells (DC) [2]. However, it has been reported that APN plays a dual role in immune system, indicating that APN possesses proinflammatory actions as a possible result of the presence of isoforms [2]. Jung et al. demonstrated that APN can promote DC activation, leading to Th1 and Th17 polarization [235].

Piccio et al. investigated the role of APN in adiponectin deficient (ADPKO) mice with EAE. These mice developed higher CNS inflammation, demyelination, and axonal injury. T cells from ADPKO mice produced higher levels of TNF-*α*, IFN-*γ*, IL-17, and IL-6 and a decreased Treg number and function, demonstrating that the absence of APN contributes to elevating the disease severity. Furthermore, the use of APN as a treatment in these animals ameliorates EAE by the increase of the number of Treg [36]. These data indicate that APN could exert beneficial effects on the treatment of MS, but information on the role of APN with the animal model of MS is insufficient and more efforts must be made in this respect.

Regarding APN serum levels in patients with MS, Kraszula et al. observed that APN is decreased in patients with RRMS compared with healthy individuals. Also, the authors found higher levels of leptin, correlating with the number of Treg; however, APN was not correlated with Treg [32]. In line with these findings, Musabak et al. also found that the APN serum levels were lower than those of healthy controls, and these levels were higher in female than in male patients with MS. Then, it is possible that APN is gender-dependent, because the same behavior is observed in healthy controls [37].

In contrast, Hietaharju et al. found higher CSF concentrations of APN and adipsin in twins with MS in remission compared with their asymptomatic twins, although these levels did not correlate with plasma levels. In this regard, the authors suggest that there is a possible intrathecal synthesis of adipokines or increased transport across the BBB following enhanced systemic production [92]. However, the sample size of this study is small; thus, there is a need for larger studies to clarify the significance of APN levels in patients with MS.

#### 3.1.3. Resistin

Resistin is a mediator of insulin resistance and is also known as ADSF (ADipocyte-Secreted Factor) or FIZZ3 (found in inflammatory zone 3) [168, 236, 237]. This adipokine has been implicated in obesity, diabetes [238], atherosclerosis [239], coronary heart disease, and cardiovascular disease [240]. Additionally, there is evidence that serum resistin levels are increased in patients with rheumatoid arthritis (RA) [241].

These adipokines are produced by adipocytes [242], intestinal epithelium, adrenal gland, and skeletal muscle [243]. It has been reported that resistin can be expressed in CNS by the pituitary gland and that its expression is age- and gender-dependent in mice [244]. Also, resistin can influence sympathetic nerve activity in CNS [245].

A few studies had attempted to discover the relationship between resistin and MS pathogenesis in patients with the disease [32–34]. In an endeavor to determine the possible association, Emamgholipour et al. observed an elevation of resistin, leptin, and visfatin levels, as well as a decrease in the FoxP3 mRNA expression of T cells. Additionally, the authors found that these adipokines are positively correlated with some inflammation mediators in healthy controls, suggesting that adipokines may play a role as inducers of proinflammatory mediators (TNF-*α*, IL-1*β*, and hs-CRP) [33]. Hossein-Nezhad et al. also found higher serum levels of resistin in patients with MS compared with the control group [34], which adds evidence to support the role of resistin in MS, although more studies are needed.

#### 3.1.4. Visfatin

Visfatin was originally known as PBEF (Pre-B cell colony-Enhancing Factor). Leukocytes, adipose tissue macrophages, hepatocytes, or skeletal muscle participates in visfatin production [246]. Visfatin is related to glucose metabolism because it can bind to and activate the insulin receptor [247].

In the area of immune activities, visfatin induces the production of IL-6, TNF-*α*, and IL-1*β* in monocytes. Moreover, visfatin increases the surface expression of costimulatory molecules CD54, CD40, and CD80 [248]. In RA, visfatin is described as a proinflammatory and destructive mediator of joint inflammation in RA [249]. Emamgholipour et al. found a positive correlation of visfatin with TNF-*α*, IL-1*β*, and hs-CRP in patients with MS [33]. Almost nothing is known about the relationship between visfatin and MS.

#### 3.1.5. Adipocyte-Fatty Acid-Binding Protein

Serum Adipocyte-Fatty Acid-Binding Protein (A-FABP) is produced by adipose tissue, monocytes, and macrophages, and its expression is enhanced by Toll-Like Receptor-2 (TLR-2) stimulation [250]. Higher levels of A-FABP have been associated with increased triglycerides, elevated fasting serum glucose, and hs-CRP in coronary artery disease [251]. Regarding MS, A-FABP levels are highest in Secondary Progressive MS (SPMS), suggesting a possible role in the pathogenesis of this disease subtype. Also, A-FABP levels are increased in patients with Pediatric-Onset MS (POMS) and may play a role in the early stages of disease [39]. Therefore, more studies are necessary on this adipokine and its relationship with MS pathogenesis in humans and the animal model to clarify the mechanisms in which A-FABP is involved.

#### 3.1.6. Adipsin

Adipsin was described as a molecular marker of obesity in rodents [252]. The function of adipsin in relation to energy homeostasis and systemic metabolism remains unknown [253]. In patients with a specific neurological diagnosis without prior selection, serum adipsin levels are correlated with CSF levels. Also, serum and CSF levels are correlated with age and are higher in patients with diabetes mellitus or hypertension. Adipsin CSF levels are correlated with inflammation mediators, but not with the presence of oligoclonal bands [93]. In a study of twins with MS, Hietaharju et al. found higher CSF concentrations of APN and adipsin in twins with MS in remission compared with their asymptomatic twins and no correlation with its plasma levels [92]. Recently, a study conducted by Natarajan et al. has provided novel insights into the impact of adipokines on MS and suggests that adipsin exerts predictive potential as a biomarker of neurodegeneration [254]. However, very little is known about the role of adipsin in MS pathogenesis; therefore, more studies are needed.

#### 3.1.7. Chemerin

Chemerin is an adipokine secreted by adipocytes and is associated with obesity, the metabolic syndrome, and insulin resistance [255]. Chemerin has been identified as a chemoattractant for Antigen Presenting Cells (APC), including DC and macrophages; in addition its receptor ChemR23 induces the release of calcium, inhibition of cAMP accumulation, and phosphorylation of p42–p44 MAP kinases [256]. In addition, chemerin is a proteolytically regulated leukocyte chemoattractant when it binds to CheMoKine-Like Receptor-1 (CMKLR-1) [94].

This adipokine is expressed in vascular Endothelial Cells in the meninges and in white-matter lesions of MS, whereas its receptor is expressed in infiltrating leukocytes, including plasmacytoid DC. These data suggest that chemerin is directly involved in the migration of peripheral cells into the CNS and that they contribute to the inflammatory process [95]. CMKLR-1 Knock-Out (KO) mice exhibit reduced symptoms of EAE [94]. It has been demonstrated that targeting with alpha-NETA (*α*-NETA), an antagonist of CMKLR1, inhibits CNS-infiltrating cells and modulates inflammation in EAE mice. However, *α*-NETA does not modify T cell proliferation [257]. In a recent study, it was found that chemerin plasma levels are higher in patients with MS with overweight or obesity compared with patients with MS and without obesity and controls [38]. These results suggest that obesity in patients with MS increases chemerin levels and causes an increase in CNS-infiltrating cells that may, in turn, contribute to disease severity.

#### 3.1.8. Omentin

Omentin is produced mainly by Visceral Adipose Tissue and its expression is reduced in obesity, insulin resistance, and type 2 diabetes. This adipokine has anti-inflammatory, antiatherogenic, anticardiovascular, and antidiabetic effects [258]. In patients with MS, omentin-1 serum levels are correlated with Bone Mineral Density (BMD) at femoral neck, total hip, osteopontin, and osteocalcin [96]. It has been reported that omentin-1 is involved with insulin activity, induces Akt phosphorylation [259], and is inversely correlated with obesity [260]. These evidences provide little knowledge; thus, more studies on these adipokines are needed to establish whether or not omentin participated in the pathogenesis of MS.

#### 3.1.9. Vaspin

Vaspin is an adipokine that is predominantly secreted by Visceral Adipose Tissue and its serum levels are increased and associated with obesity and impaired insulin sensitivity. To the contrary, its correlation is abrogated in type 2 diabetes and its levels are higher in females as compared with males [261]. Vaspin has been poorly studied in patients with MS. In this regard, Assadi et al. did not find any correlation between vaspin and age, BMI, biochemical, and BMD measurements in patients with MS [96].

**Figure 1:**
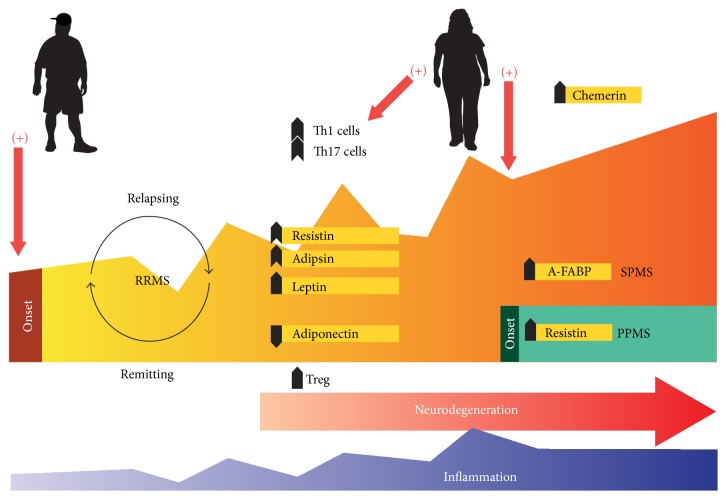
An integrative view of the possible involvement of some adipokines present in patients with multiple sclerosis (MS) at different disease stages. Obesity during adolescence constitutes a relevant risk factor for the later development of MS. Adult obesity negatively affects evolution of the disease and response to treatment in patients with MS. During relapsing-remitting stages, a decrease in adiponectin has been reported as well as, concomitantly, an increase in resistin, adipsin, and leptin concentrations (in serum or CSF or both). Additionally, A-FABP is increased in patients with SPMS, and resistin is also increased in patients with PPMS. Given that inflammation occurs at a variable intensity from the onset of the disease and that neurodegeneration process starts after disease initiation, in this context the adipokines produced by lipid tissue constitute an additional element in the neuroimmunomodulation complex of organisms with MS (see text for further explanation); thus the adipokines produced constitute an additional element in the neuroimmunomodulation complex of organisms with MS (see text for further explanation). RRMS, Remittent Recurrent Multiple Sclerosis. SPMS, Secondary Progressive Multiple Sclerosis. PPMS, Primary Progressive Multiple Sclerosis. CSF, CerebroSpinal Fluid. Resistin (serum) [32–34]. Adipsin (in CSF) [35]. Leptin (serum and CSF) [35]. Adiponectin (in serum) [32, 36, 37]. Chemerin (serum) [38]. A-FABP, Adipocyte-Fatty Acid-Binding Protein (in serum) [39].

In summary, dysregulated adipokines can be involved in the pathophysiology of MS, increasing the risk of the disease development after obesity, during adolescence or the early-adult stage, as well as influencing elements that affect disease evolution and treatment response for obese adults with MS (Figure 1). Undoubtedly, there remains such research to be conducted with regard to these aspects.

## 4. Transplantation of Adipose Tissue-Mesenchymal Stem Cells as Therapy for MS

Mesenchymal Stem Cells (MSC) are a pleiotropic population of precursor cells that are self-renewing and capable of differentiating into canonical cells of the mesenchyme, including adipocytes, chondrocytes, and osteocytes [262]. Due to their immunomodulatory and neuroprotective effects, Adipose Tissue-Mesenchymal Stem Cells (AT-MSC) may be proper candidates for stem cell-based MS therapy. The intraperitoneal (i.p.) route exerts a more pronounced effect on maintaining splenic CD4+CD25+FOXP3+ T cell population and increasing IL-4 secretion. In addition, i.p. injection of cells resulted in lower IFN-*γ* secretion and reduced cell infiltration in brain more effectively as compared with the intravascular (i.v.) route [263].

In mice with established EAE,* in vivo* infusion of wild-type Adipose Stromal/Stem Cells (ASC) significantly ameliorated the disease course, autoimmune-mediated demyelination, and cell infiltration through regulation of the inflammatory responses. However, mice treated with autologous ASC exhibited no therapeutic improvement in terms of disease progression [264].

The preclinical efficacy of AT-MSC obtained from the SJL/JCrl mouse strain (SJL-AdMSC) has been assessed by autologous transplantation in RR-EAE-induced SJL mice (a well-established mouse model for the study of RRMS) by ameliorating the RR-EAE course, suggesting that these could modulate disease progression [265].

The therapeutic efficacy of ASC isolated from lean subjects (BMI < 25, lnASC) and subjects with obesity (BMI > 30 in ObASC) were determined in murine EAE. Compared with EAE disease-modifying effects of lnASC, ObASC consistently failed to alleviate clinical symptoms or to inhibit inflammation in the CNS. When activated, ObASC expressed higher mRNA levels of several proinflammatory cytokines compared with InASC. Additionally, Conditioned Media (CM) collected from ObASC markedly enhanced T cell proliferation and differentiation, whereas CM from lnASC did not. These results indicate that obesity reduces, or eliminates, the anti-inflammatory effects of human ASC; therefore, they may not be a suitable cell source for the treatment of autoimmune diseases. The data suggest that donor demographics may be particularly important when identifying suitable stem cells for treatment [266].

The majority of Mesenchymal Stem Cells (MSC) clinical trials are currently in phase 2 of development, during which safety and tolerability of treatment continue to be evaluated (Table 3). These works include patients with median or high Expanded Disability Status Score (EDSS) and who are mainly in the SPMS stage. Sample size is reduced and the protocol of administration is variable, both in number of cells transplanted and in the administration pathway (mainly intravenously or intrathecally). It appears to be that, in the majority of cases, there is a decrease in the EDSS index after transplantation; however, it is necessary to follow up patients for a longer time period (>6 months) and to be more accurate with respect to the possible improvement achieved. The results are also highly variable, as well as the adverse events presented (see summary, Table 3). Consequently, although AT-MSC transplantation can be regarded as a potential source of treatment for MS, several studies now at clinical stages need to see whether these show a real benefit in practice, particularly in MS progressive stages (PPMS and SPMS). These works will contribute to the design of future trials conducted to establish whether MSC transplantation comprises an effective therapy for patients with MS.

**Figure 2:**
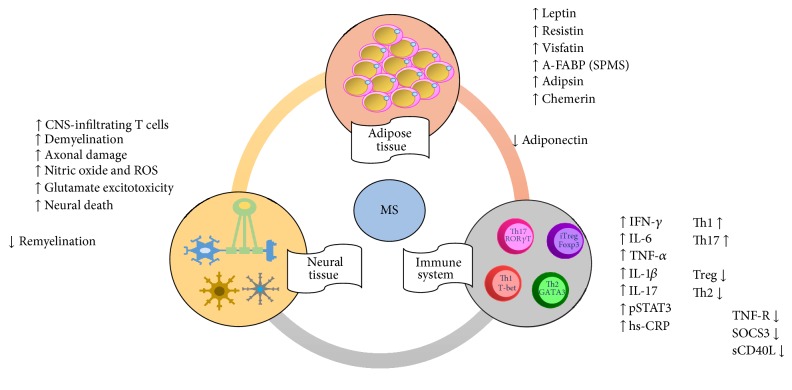
Cross talk among immune, neural, and adipose tissues. Adipocytes release leptin, resistin, and visfatin, which induce a low-grade inflammatory state in patients with multiple sclerosis (MS) with obesity. T cells migrate into the Central Nervous System (CNS), with Th1/Th17 cell release of proinflammatory cytokines, which promote the inflammatory status, and Th2/Treg release of anti-inflammatory cytokines, which contributes to modulating the severity of multiple sclerosis (MS). Neurons and oligodendrocytes are those mainly affected in MS. Axons are demyelinated by cell- and molecular-mediated mechanisms. Antigen presentation between microglia and CNS-infiltrating T cells induces a proinflammatory positive feedback loop. Astrocytes costimulate CNS-infiltrating T cells through CD24 expression.

In conclusion, since the discovery of the remarkable properties of adipose tissue the possible link between MS and obesity has been rendered even more interesting. Several evidences on the possible link between obesity and the pathogenesis of MS were discussed. Although it is well-known that CNS and immune system cells are involved in the pathogenesis of MS, adipokines comprise the possible cross talk between them (Figure 2). Therefore, it will be also relevant to explore the role of neuropeptides, like NPY, leptin, ghrelin, and other proteins, in relation to feeding behavior, whose mechanisms are regulated by orexigenic and anorexigenic hypothalamic neurons, because an adequate regulation of this neural circuitry will be related to an improvement in the inflammatory response and survival in MS. Finally, it seems to be more clear that once MS is initiated, obesity can contribute to increasing disease severity by negatively influencing disease progress and treatment response, but, also, obesity in early life (mainly during adolescence) is highly relevant as a susceptibility factor and causally related increased risk for late MS development.

## Figures and Tables

**Table 1:** Cell types affected in the experimental autoimmune encephalomyelitis (EAE) mouse model or in patients with multiple sclerosis (MS).

Adipokine	EAE	Multiple sclerosis	Reference
Leptin		↓ IFN-*γ*- and IL-17-secreting T cells in patients treated with Metformin ↓ TNF-*α*- and IL-6-secreting T cells in patients treated with Pioglitazone ↑ CD4+CD25+FoxP3 Treg in patients treated with Metformin and Pioglitazone	[72]
		↓ NK cells, ↓ B cells, ↓ CD4+DR+ T cells, and ↓ CD4+CD45RA+ T cells in patients treated with GA	[73]
		↓ nTreg cells in patients with RRMS	[74]
	↓ CD4+ T cells, CD8+ T cells, and CD11b+Gr1+ granulocytes in ENdothelial leptin receptor-specific Knock-Out (ENKO)		[32]

Adiponectin	Adiponectin treatment is associated with increase in Treg		[36]

**Table 2:** Adipokines involved in the experimental autoimmune encephalomyelitis (EAE) mouse model or in patients with multiple sclerosis (MS).

Adipokine		EAE		Multiple sclerosis
Leptin	**↑**	In serum before clinical onset [75] Transport in hippocampus and cervical spinal cord in early stage [77] In females rather than in males in pup mice and decrease in time-dependent manner [57] ↑ IFN-*γ* and TNF in Histidine DeCarboxylase-deficient (HDC−/−) mice [78] In Endothelial Leptin receptor-specific Knock-Out (ELKO) [80] Microvascular leptin receptors [80]	**↑**	In patients [32, 39], in CSF and serum [35] during remission [76] ↑ pSTAT3 and ↓ SOCS3 in patients with RRMS on relapsing [76] ↓ sCD40L and ↓ TNF-R in patients treated with GA [73] In IFN-*β*-treated patients [79] In females [81] and pregnant females with MS [82] ↑ Leptin receptor in CD8+ T cells in monocytes from patients with RRMS in relapse rather than remission [76] ↑ Leptin receptor (ObR) in T cell lines after activation with human Myelin Basic Protein (hMBP) [35]

Leptin	**↓**	In triterpene-pretreated EAE mice [83] ↓ IL-6 in EAE mice with caloric restriction [84]	**↓**	In patients treated with Metformin and Pioglitazone [72] In postdelivery females with MS [82] After IFN-*β* treatment [85] and ↓ IL-6 in patients with SPMS treated with IFN-*β* [86] In untreated or methylprednisolone-treated patients [79]

Leptin		Negatively correlates with clinical score in females [57] Correlates with disease susceptibility, reduction in food intake, and decrease in body weight [75] Blockade of leptin with antileptin or soluble mouse receptor chimera (ObR:FC) improves clinical score, reduces disease relapses, inhibits T cell proliferation, and induces Th2 cytokine profile [88]		Negatively correlates with testosterone in males [87] Positively correlates with BMI in females [87] Correlates with disease duration, but not with EDSS, in females [81] ObR in plasma are similar between patients and controls [79] Correlates in serum with IFN-*γ* in CSF [35] Inversely correlates with Treg [35] Correlates with BMI in patients with low and intermediate, but not with high, EDSS [89] Induces IFN-*γ* in PBMC from patients with MS in relapse, but not in remission [90] Induces TNF-*α*, IL-6, and IL-10 production in PBMC from relapsing patients with MS, but not in remission [91]

Adiponectin		As treatment, ameliorates EAE in ADPKO mice [84]	**↓ **	In patients with MS [37] In patients with RRMS [32]
			**↑**	In patients treated with Metformin and Pioglitazone [72] In CSF from twins with MS [92] ↓ IL-6 in EAE mice with caloric restriction [84]

Resistin			**↑**	In patients with MS and in patients with RRMS [32] ↑ TNF-*α*, ↑ IL-1*β*, and ↑ hs-CRP in patients with MS [34] and in patients with PPMS [33]

Visfatin			**↑**	In patients [33]

A-FABP			**↑**	In patients with SPMS and in pediatric-onset patients [39]

Adipsin			**↑**	In CSF from twins with MS [92] In serum and CSF in individuals with overweight/obesity and in patients suffering from hypertension or diabetes mellitus [93] Correlates in serum and CSF and is gender-independent [93] In CSF does not correlate with oligoclonal-band presence [93]

Chemerin	**↑**	In CNS of mice [94] It is present in intralesional cerebrovascular endothelial cells, and its receptor is expressed in CNS-infiltrating leukocytes [95]	**↑**	In patients with MS with overweight/obesity [38]

Omentin-1				Correlates with BMD at femoral neck, total hip, osteopontin, and osteocalcin [96]

Vaspin				Does not correlate with age, BMI, biochemical, and BMD measurements in patients with MS [96]

**Table 3:** Studies in human therapy for MS by transplants of adipose tissue-mesenchymal stem cells.

MS form	Sample size	Dosage and administration	Changes in EDSS	Adverse events	Results	Ref
SPMS	10	1.6 × 10^6^ cells/Kg Intravenously	Initial 6.1 decrease after treatment	Macular rash, scalp pruritus, upper-respiratory tract infection, and urinary-tract infection	No changes in the posttreatment period for T cell subset counts (CD3, CD4, CD8, CD19, and CD56) Decrease of T1 hypointense lesion volume after treatment Increase in optic nerve area and reduction in visual evoked response latency	[97]

SPMS and PRMS	25 (23/2)	2.95 × 10^7^ cells Intrathecally	Initial 5.5 to 7 decrease or stability after treatment in 68% of patients	N/A	MRI score decrease in 68% of patients Increase of IL-6 gene expression No changes in IL-4, IL-6, IL-10, IFN-*γ*, and TGF-*β* gene expression	[98]

SPMS and RRMS	10 (9/1)	(a) High 1 × 10^8^ cells (b) Medium 3.2–5.2 × 10^7^ cells (c) Low 1.1–1.5 × 10^6^ cells Intrathecally and intracisternally	Initial 4.0 to 7.5 decrease after treatment	Transient encephalopathy with seizures in one patient	Clinical but not radiological efficacy Improvement in 5/6 and worsening in 1/6 patients New or enlarging lesions in 5/7 and Gadolinium (Gd+) enhancing lesions in 3/7 patients	[99]

MS	15	6.32 × 10^7^ cells Intrathecally	Initial 6.7 decrease after treatment	Transient fever and headache	Increase of CD4+CD25+ Treg Decrease of CD40+, CD83+, CD86+, and HLA-DR on myeloid dendritic cells	[100]

MS	1	N/A	N/A	The patient development headache, nausea, vomiting, and difficulty of ambulation	Accumulation of CD-68 positive myelin-laden macrophages and T cells Extensive demyelination in a pattern typical of multiple sclerosis in brain biopsy	[101]

PRMS = progressive relapsing multiple sclerosis.

## Abbreviations

APN: Adiponectin
BBB: Blood Brain Barrier
CNS: Central Nervous System
CSF: CerebroSpinal Fluid
EDSS: Disability Status Scale
EC: Endothelial Cells
EAE: Experimental Autoimmune Encephalomyelitis
GA: Glatiramer Acetate
GS: Glutamine Synthetase
GDH: Glutamate DeHydrogenase
IGF-1: Insulin-Like Growth Factor-1
ICAM-1: IntraCellular Adhesion Molecule-1
MOG: Myelin Oligodendrocyte Glycoprotein
MS: Multiple Sclerosis
OPC: Oligodendrocyte Precursor Cells
PERK: Pancreatic Endoplasmic Reticulum Kinase
POMS: Pediatric-Onset MS
PBMC: Peripheral Blood Mononuclear Cells
PPAR-*γ*: Peroxisome Proliferator-Activated Receptor gamma
PD-L1: Programmed Death-Ligand-1
PD1: Programmed Death-1
SPMS: Secondary Progressive MS
A-FABP: Adipocyte-Fatty Acid-Binding Protein
SAT: Subcutaneous Adipose Tissue
VAT: Visceral Adipose Tissue.

## Additional Points

Apologies are due to authors whose works have not been reviewed and to those whose papers have not received the emphasis that they merit. The authors also apologize to authors whose work has not been appropriately cited due to space limitations and/or to limitations of the present paper's authors' knowledge.
